# Design of a modular solver - Excel spreadsheet decision-support tool for calibrated nonlinear farm optimization in small holder farm systems

**DOI:** 10.1016/j.mex.2026.103911

**Published:** 2026-04-15

**Authors:** Welcome Zimuto, Samuel Ahado, Tomas Ratinger, Jindrich Spicka

**Affiliations:** a Faculty of Tropical AgriSciences, Czech University of Life Sciences Prague, Czech Republic; b Faculty of Economics and Management, Czech University of Life Sciences Prague, Czech Republic; c Department of Strategic Studies, Technology Centre Prague, Czech Republic

**Keywords:** Nonlinear optimization, Decision-support, Smallholder farm systems

## Abstract

This study presents the design of a modular General Algebraic Modelling System (GAMS) solver integrated with an Excel spreadsheet as part of a decision-support system for implementing calibrated nonlinear farm optimization models under data-limited smallholder farm conditions. The study does not claim methodological innovation in nonlinear Positive Mathematical Programming or causal inference regarding farmer behavior. Instead, the optimization model is treated as a computational component embedded within a broader digital decision-support framework. The model system separates data input, scenario execution, model calibration, and output interpretation into modular components, enabling repeated simulations without requiring model modification. A baseline linear programming (LP) model is sequentially calibrated using dual values to recover implicit costs. The quadratic cost parameters are specified such that base-year replication matches the observed values exactly. The model includes automated looping routines that allow systematic price perturbation and supply response analysis. Validation employs base-year replication metrics, convexity checks, and solver optimality checks. Overall, the model provides a transferable framework that embeds nonlinear farm optimization into extension-relevant decision-support environments while maintaining computational transparency and reproducibility.

• The design decouples the input data from model execution, enabling rapid simulations without interfering with the underlying GAMS code.
• The calibration process is sequential, utilizing dual values to recover implicit costs, ensuring the model exactly replicates observed smallholder data behavior in data-limited smallholder farm environments.
• The automated looping routines enable high-speed, systematic price sensitivity and supply response analysis, providing a transparent and reproducible architecture for agricultural extension optimisation advice and ex-ante measures

## Graphical abstract

**Figure:**
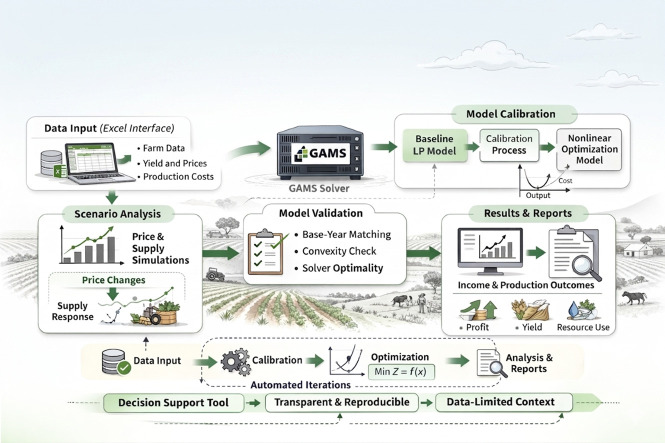

**Specifications**

**Table:** 

Subject area	Agricultural and Biological Sciences
Specific subject area	Agricultural Economics
Name of method	Farm Systems Nonlinear Optimization Modeling
Name and reference of the original method	Positive Mathematical Programming Modeling by [4]
Resource availability	Latex code and reproducibility file attached

## Background

This study contributes to the literature on digital decision-support systems for agriculture by designing and implementing a spreadsheet–solver–based model that enables nonlinear farm optimization under data-scarce conditions for smallholders. Rather than advancing Positive Mathematical Programming (PMP) methodology [[1], [2], [3]] itself, the contribution lies in the integration, calibration workflow, and system-level implementation that allows farm-level optimization models to be operationalized within a low-cost, reproducible, and extension-relevant computing environment. In this instance, low cost refers to the data requirements and expertise needed to implement the architecture, rather than the costs associated with acquiring software licenses Table 2.

Below, we highlight how the decision-support system is structured

○ spreadsheet-based data entry with a nonlinear optimization engine via automated data exchange;
○ a calibration and scenario workflow that enables repeated,
○ reproducible simulations without manual code model rewriting;

The above are set to demonstrate how farm-level optimization outputs (activity levels, shadow prices, constraint utilization) can be structured for advisory interpretation rather than standalone economic analysis.

The methodological approach presents a modular computational framework for embedding a calibrated GAM Solver Excel Spreadsheet decision-support environment. The objective is to operationalize Positive Mathematical Programming (PMP) in a reproducible framework suitable for smallholder agricultural systems. Unlike theoretical contributions to nonlinear programming, this study focuses on system-level design, workflow automation, and separation of data, model, and scenario components.

The implementation is sequentially administered in four stages, highlighted as follows;

○ A baseline Linear Programming Model
○ A calibration linear Model
○ A non-linear PMP model
○ Scenario and policy simulations

In this model, the core sets include inputs (INPUT), resources (ROWS), farm activities (ACT), and outputs (OUTPUT). Crop outputs and non-agricultural outputs are considered separately. Base year activity levels are denoted as X0(c). The parameters include resource endowments (RHS), technical coefficients (TECHCO), input prices (W), crop yields [5] (YIELD), and output prices (W). To construct the model matrix, we use the technical coefficient matrix.

## Method details

### System architecture

A decision support system (DSS) is proposed, comprising a modular computational workflow that separates data handling, optimization, scenario analysis, and result interpretation. Separation of these components enables reproducibility, adaptability, and transparency under different farm system contexts. The Modular Decision Support System consists of four independent modules:

i. Data Layer
ii. Optimization Layer [6]
iii. Scenario Engine [7]
iv. Output Layer

This separation ensures transparency, reproducibility, and transferability.

### Step 1 – data preparation and organization

Prepare farm survey data and organize it into an Excel workbook with the following standardized tabs in one spreadsheet

Perform the following checks

○ Non-negativity enforcement
○ Unit harmonization
○ Resource balance validation
○ Missing value checks
○ Data is transferred to GAMS using GDXXRW to generate a GDX file.

**Fig. 1:**
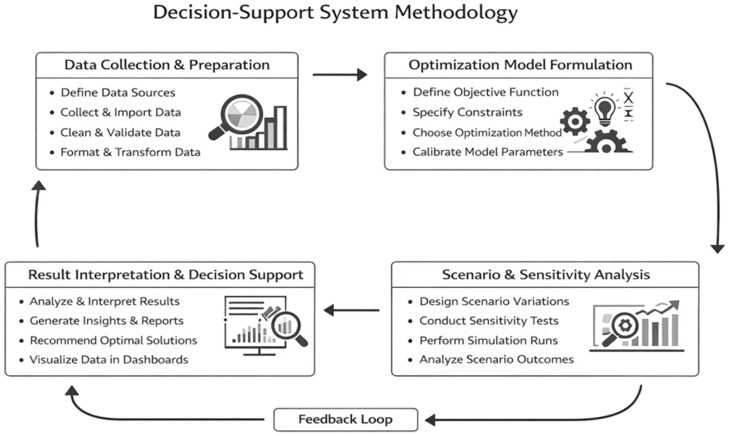
Model Design Framework.

The proposed model framework system as shown in Fig. 1 below, illustrates the flow of data Table 1.

**Table 1:** Excel Sheet Parameters.

Sheet	Description	Units
ACTIVITIES	Activity identifiers	–
TECHCO	Technical coefficients (a_rc)	Input per ha
RESOURCES	Resource endowments (RHS_r)	Resource units
PRICES	Output prices (p_c)	USD/unit
COSTS	Input costs	USD/unit
BASEYEAR	Observed activity levels (X0_c)	Ha

Farm-level data is collected via a structured survey and entered into a standardized Excel spreadsheet. The Excel spreadsheet environment serves as the primary data layer because it is accessible, has low technical barriers, and is familiar to researchers, agricultural practitioners, and extension officials. The data preprocessing includes aggregating activity-level coefficients, validating resource endowments, and performing consistency checks for model execution.

### Step 2: construction of revenue and cost coefficients

The gross revenue per hectare for crop cis defined as the product of the output price and yield:

(1) $$\text{RE}V_{c}=P_{c}\cdot Y_{c}$$

Where:

○ $\text{RE}V_{c}$= Revenue per hectare for crop c
○ $P_{c}$= Market price of crop c(e.g., $/ton)
○ $Y_{c}$= Yield of crop c(e.g., tons/ha)

This formulation assumes constant prices and yields within the production period. If prices or yields are stochastic, expected values should be used. Total variable input cost per hectare for crop cis calculated as the weighted sum of input requirements across all input factors f:

(2) $$\text{COS}T_{c}=\sum_{f=1}^{F}a_{fc}\cdot w_{f}$$

Where:

○ $\text{COS}T_{c}$= Total input cost per hectare for crop c
○ $a_{fc}$= Technical coefficient representing the quantity of input frequired per hectare of crop c
○ $w_{f}$= Unit price of input factor f
○ F= Total number of input factors

The gross margin coeffic ient is defined as follows:

(3) $$\pi_{c}=\text{RE}V_{c}-\text{COS}T_{c}$$

Where:

○ $\pi_{c}$= Net Return (gross margin) per hectare of crop c

The matrix form for the optimization PMP model is implemented in linear programming form:

(4) REV=P∘Y

(5) $$\text{COST}=A^{⊤}w$$

Where:

○ $A=[a_{fc}]$is the input–output coefficient matrix
○ wis the vector of input prices
○ ∘denotes element-wise multiplication

The revenue and cost components form the objective coefficient row of the linear model. These revenue and cost components form the objective coefficient row of the linear programming model.

### Step 3: construction of baseline linear programming model

The baseline LP model maximizes the total gross margin, also referred to as profit and is denoted with the equation below;

(6) $$\text{max}\sum_{c}\pi_{c}X_{c}$$

Subject to:

(7) $$\sum_{c}a_{rc}X_{c}\leq RHS_{r}$$

(8) $$X_{c}\geq 0$$

Where:

○ $X_{c}$: hectares allocated
○ $\pi_{c}=p_{c}y_{c}-VC_{c}$: gross margin
○ $a_{rc}$: technical coefficient
○ $RHS_{r}$: resource endowment

Note that the LP model's costs are linear and its marginal costs are constant; therefore, the linear programming solution produces corner solutions that select only the most profitable agricultural activities. The shadow prices are obtained from the dual values of the resource constraints, and they represent the marginal resource values.

### Step 4: calibration procedure

To recover implicit marginal costs and ensure exact base-year replication, the following calibration constraint is imposed for each activity c:

(9) $$\begin{array}{cccc} & X_{c}\leq X_{c}^{0}\left(1+\varepsilon \right) & & \end{array}$$

where:

○ $X_{c}$= decision variable representing activity level c
○ $X_{c}^{0}$= observed base-year level of activity c
○ ε= small positive perturbation parameter (e.g., $10^{-4}$), where ε = 0.0001

The model is solved as linear and maximizes the same objective function. Introducing μ_c as a dual variable for the calibration constraint, the first-order conditions imply that μ_c captures the implicit marginal costs that prevent expansion beyond observed activity levels.

### First-Order condition from the calibrated linear model

The calibration multipliers are used to construct the nonlinear cost function by exploiting the first-order optimality condition of the constrained linear program:

(10) $$\begin{array}{cccc} & \pi_{c}=\sum_{r}\lambda_{r}a_{rc}+\mu_{c} & & \end{array}$$

where:

○ $\pi_{c}$= observed gross margin per unit of activity c
○ $\lambda_{r}$= shadow price (dual value) of resource constraint r
○ $a_{rc}$= technical coefficient representing the use of the resource rby activity c
○ $\mu_{c}$= calibration multiplier (dual value of the calibration constraint)

Equation () represents the **Karush–Kuhn–Tucker (KKT) first-order condition** [8] of the calibrated linear program. KKT states that the observed gross margin $\pi_{c}$ equals the sum of implicit marginal resource costs $\sum_{r}\lambda_{r}a_{rc}$ plus the calibration multiplier $\mu_{c}$, which captures unobserved or implicit marginal costs preventing expansion beyond the base-year activity level.

The multiplier $\mu_{c}$ measures the deviation between observed marginal revenue and the marginal opportunity cost of resources and is subsequently used to construct the quadratic cost curvature parameter in the PMP formulation. Thus, μ_c captures the implicit marginal costs that prevent expansion beyond observed activity levels. These multipliers are used to construct a nonlinear cost function.

### Derivation of nonlinear cost parameters

The quadratic cost function for the activity c is defined as:

(11) $$\begin{array}{cccc} & C_{c}\left(X_{c}\right)=\frac{1}{2}\;\beta_{c}X_{c}^{2} & & \end{array}$$

where:

○ $C_{c}\left(X_{c}\right)$= total nonlinear cost associated with activity
○ $X_{c}$= activity level (e.g., hectares allocated)
○ $\beta_{c}$= curvature parameter governing the rate of increasing marginal cost

The quadratic cost curvature parameter for each activity cis computed as:

(12) $$\begin{array}{cccc} & \beta_{c}=\frac{COST_{c}+\mu_{c}\;}{X_{c}^{0}} & & \end{array}$$

where:

○ $\beta_{c}$= curvature parameter of the quadratic cost function for activity c
○ $COST_{c}$= observed average variable cost per hectare in the base year
○ $\mu_{c}$= dual value (calibration multiplier) associated with the calibration constraint for activity c
○ $X_{c}^{0}$= observed base-year activity level for activity c

This approach provides confirmation that the marginal cost in the base year equals the observed marginal revenue, ensuring exact replication of the base solution.

### Step 5: nonlinear PMP construction

After calibrating the linear programming benchmark, we now add a quadratic cost function to recover implicit marginal costs and achieve an exact match with observed production patterns. This non-linear profit-maximization framework features conventional resource constraints and curvature parameters associated with unobserved adjustment costs and behavioral inertia. Below is the model structure as illustrated in the preceding section

The objective of the calibrated nonlinear PMP model is:

(13) $$\begin{array}{cccc} & \text{max}\Pi^{\text{PMP}}=\sum_{c}\pi_{c}X_{c}-\sum_{c}\frac{1}{2}\beta_{c}X_{c}^{2} & & \end{array}$$

where:

○ $\Pi^{\text{PMP}}$= total farm profit under the nonlinear PMP specification
○ $\pi_{c}$= observed gross margin per unit of activity c
○ $X_{c}$= decision variable (activity level)
○ $\beta_{c}$= curvature parameter of the quadratic cost function

Subject to resource constraints:

(14) $$\begin{array}{cccc} & \sum_{c}a_{rc}X_{c}=RHS_{r}\forall r\in R_{E} & & \end{array}$$

(15) $$\begin{array}{cccc} & \sum_{c}a_{rc}X_{c}\leq RHS_{r}\forall r\in R_{I} & & \end{array}$$

(16) $$\begin{array}{cccc} & X_{c}\geq 0\forall c & & \end{array}\;$$

where:

○ $a_{rc}$= technical coefficient of resource rused by activity c
○ $RHS_{r}$= available endowment of resource r
○ $R_{E}$= set of equality-constrained resources
○ $R_{I}$= set of inequality-constrained resources

### First-Order optimality condition

The first-order condition (KKT condition) for each activity cis:

(17) $$\begin{array}{cccc} & \pi_{c}-\beta_{c}X_{c}-\sum_{r}\lambda_{r}a_{rc}=0 & & \end{array}$$

where:

○ $\lambda_{r}$= shadow price of resource constraint r

The quadratic term $\frac{1}{2}\beta_{c}X_{c}^{2}$introduces increasing marginal costs, such that:

(18) $$\frac{\partial }{\partial X_{c}}\left(\frac{1}{2}\beta_{c}X_{c}^{2}\right)=\beta_{c}X_{c}$$

This prevents corner specialization typical of linear programming models and produces smooth interior solutions.

### Base-Year replication condition

Substituting the observed base-year activity level $X_{c}^{0}$into the first-order condition yields:

(19) $$\beta_{c}X_{c}^{0}=COST_{c}+\mu_{c}$$

where:

○ $COST_{c}$= observed average variable cost
○ $\mu_{c}$= calibration multiplier from the constrained LP [9]

The above equation condition ensures that the nonlinear model exactly reproduces the observed base-year allocation. Therefore, the nonlinear model reproduces the observed base-year allocation exactly without calibration constraint.

### Step 6: interpretation of model outputs

The main outputs of the model include objective function values, optimal allocations of agricultural and non-agricultural activities, shadow prices for binding constraints, and utilization rates for each resource. The DSS is designed to ensure that comparative analysis can generate prescriptive farm recommendations to agricultural authorities, which can be used in extension advice. Results displayed in the model summaries in GAMS can be exported back to Excel spreadsheets or tables and, further, converted to dashboards for easy ex-ante policy interpretation [10].

Farm activity levels (X) represent optimal hectares allocated to each activity. The shadow prices (constraints multipliers) represent the marginal values of relaxing resource constraints. Calibration multipliers (μ_c) reflect the implicit marginal costs embedded in the base-year data. The curvature parameters (β_c) determine the slope of marginal cost functions and, therefore, the responsiveness of the supply. Compared with the LP model, the PMP model avoids extreme specialization, produces interior solutions, and yields smooth supply responses (Table 2, Table 3).

**Table 2:** Optimization software tools comparison.

Feature	Excel-GAMS Modular Tool	Pyomo + Ipopt (Open Source)
Financial Cost	Commercial but also offers free Academic/Community/Demo Licenses	Free and open source
Technicalrequirements	Relies on familiar Excel Logic and automated binary (GDX) data conversion	Requires proficiency in Python and environment management
Learning Curve	Beginners and non-programmers can leverage the GAMS Model Library and support forums to construct the model	Requires mastering object- oriented programming and library dependencies
Run time and generation	Uses relational algebra, which is significantly faster instance generation than other tools such as Pyomo+Ipopt	Python overhead can slow run time and generation for complex sets
Transparency	High due to reliance on algebraic syntax	Moderate due to imperative code heaviness

**Table 3:** Implementation transparency table illustrating the design components and corresponding specifications.

Component	Specification
Modeling platform	GAMS
Data exchange	GDXXRW / GDX
LP solver	CPLEX
NLP solver	CONOPT
Optimality tolerance	1e−6
Feasibility tolerance	1e−6
Convexity requirement	β_*c* > 0
Execution mode	Batch loop
Runtime reporting	Seconds per scenario

### Method validation

Validation is confirmed by the following

○ LP specialization occurs
○ Calibration reproduces base year farm activity levels
○ PMP replicates the base solution closely as the observed solution
○ All parameters (β_c) are positive
○ Simulation responses are smooth and economically interpretable

### Limitations

The DSS has the following limitations:

○ It is static and deterministic
○ Does not model stochastic shocks
○ Does not incorporate risk
○ Does not capture intertemporal dynamics
○ Assumes accurate base-year observations

## Ethics statements

This research did not involve experiments on humans or animals. Therefore, ethical approval on human and animal subjects was not required. The study was conducted in accordance with institutional guidelines and international research practice norms.

## Declaration of competing interest

The authors declare that they have no known competing financial interests or personal relationships that could have influenced the work reported in this paper.

## Data Availability

Data will be made available on request.

## Appendix Supplementary materials

Image, application 1
