# Supplementary material for: Design of a modular solver - Excel spreadsheet decision-support tool for calibrated nonlinear farm optimization in small holder farm systems
Source: MethodsX. 2026 Apr 15;16:103911. doi: 10.1016/j.mex.2026.103911 (PMC13191255; doi:10.1016/j.mex.2026.103911)
Supplement: Supplementary file 1 [file mmc1.docx]

Reproducibility and Model Execution Guide

# Overview

This document provides concise, step-by-step instructions for reproducing and executing the nonlinear farm optimization model implemented in the General Algebraic Modeling System (GAMS) integrated with spreadsheet-based data input. It is designed as a standalone reproducibility guide accompanying a MethodsX methods article and is intentionally limited to approximately two journal pages.

# Software and File Requirements

To execute the model, the following components are required:

- General Algebraic Modeling System (GAMS) with support for nonlinear programming - A nonlinear solver compatible with quadratic objective functions (e.g., CONOPT)
- Spreadsheet software (Microsoft Excel or equivalent)
- The GAMS model file (.gms) - The Excel data file (.xlsx) containing all model inputs

All files should be in the same working directory, or file paths should be updated consistently in the GAMS code.

# Spreadsheet Data Preparation

All model inputs are supplied through a structured Excel spreadsheet, which serves as the sole data interface. The spreadsheet must allow users to modify data and scenarios without editing the GAMS model code, supporting transparency and reproducibility.

# Importing Data into GAMS

Data are imported using the GDXXRW utility, which converts Excel ranges into a GDX file. The GDX file is then read into GAMS, where all sets and parameters are loaded automatically. Display statements included in the code allow users to verify successful data import before model execution.

# Linear Programming Base Model Execution

Model execution begins with a linear programming (LP) base model that maximizes farm profit subject to observed resource constraints. This step establishes baseline activity levels and shadow prices, which provide a benchmark for calibration and nonlinear model construction.

The LP solution is stored internally for comparison with subsequent model stages.

# Calibration Step

Calibration constraints restrict activity levels to remain within a small perturbation of observed base-year values. Solving the calibration model yields marginal values for these constraints, which are subsequently used to parameterize the nonlinear cost function in the PMP model. This step ensures consistency between observed data and optimized solutions.

# Nonlinear PMP Model Solution

The Positive Mathematical Programming (PMP) model replaces the linear objective function with a quadratic objective function that captures diminishing marginal returns. The nonlinear model is solved using an NLP solver, producing a calibrated baseline solution. Solver convergence and feasibility should be verified using the GAMS listing file.

# Scenario and Sensitivity Analysis

Scenario analysis is implemented through an automated loop that perturbs selected parameters, such as output prices. For each iteration, the PMP model is re-solved, and results are stored. This approach enables systematic sensitivity analysis without altering model structure.

# Output Review and Reproducibility Notes

Model outputs, including activity levels and diagnostic indicators, are stored in structured parameters and can be inspected directly in GAMS or exported back to Excel. The model is deterministic: identical inputs yield identical outputs. Reproducibility depends on consistent spreadsheet structure, solver settings, and parameter definitions.

# Intended Use

These instructions are intended for researchers, reviewers, and practitioners seeking to reproduce or adapt the model for exploratory and advisory analysis in smallholder farming systems.
